# Na^+^_i_,K^+^_i_-Dependent and -Independent Signaling Triggered by Cardiotonic Steroids: Facts and Artifacts

**DOI:** 10.3390/molecules22040635

**Published:** 2017-04-14

**Authors:** Sergei N. Orlov, Elizaveta A. Klimanova, Artem M. Tverskoi, Elizaveta A. Vladychenskaya, Larisa V. Smolyaninova, Olga D. Lopina

**Affiliations:** 1 Laboratory of Biological Membranes, Faculty of Biology, M.V. Lomonosov Moscow State University, 1/12 Leninskie Gory, Moscow 119234, Russia; klimanova.ea@yandex.ru (E.A.K.); garretam@ya.ru (A.M.T.); eavlad@list.ru (E.A.V.); smolyaninovalarisa1@gmail.com (L.V.S.); od_lopina@mail.ru (O.D.L.); 2 Department of Medical Biology, Siberian Medical State University, Tomsk 634055, Russia; 3 Department of Sports Tourism Sports Physiology and Medicine, National Research Tomsk State University, Tomsk 634050, Russia

**Keywords:** cardiotonic steroids, Na^+^,K^+^-ATPase, transcription, translation, proliferation, adhesion, cell death

## Abstract

Na^+^,K^+^-ATPase is the only known receptor of cardiotonic steroids (CTS) whose interaction with catalytic α-subunits leads to inhibition of this enzyme. As predicted, CTS affect numerous cellular functions related to the maintenance of the transmembrane gradient of monovalent cations, such as electrical membrane potential, cell volume, transepithelial movement of salt and osmotically-obliged water, symport of Na^+^ with inorganic phosphate, glucose, amino acids, nucleotides, etc. During the last two decades, it was shown that side-by-side with these canonical Na^+^_i_/K^+^_i_-dependent cellular responses, long-term exposure to CTS affects transcription, translation, tight junction, cell adhesion and exhibits tissue-specific impact on cell survival and death. It was also shown that CTS trigger diverse signaling cascades via conformational transitions of the Na^+^,K^+^-ATPase α-subunit that, in turn, results in the activation of membrane-associated non-receptor tyrosine kinase Src, phosphatidylinositol 3-kinase and the inositol 1,4,5-triphosphate receptor. These findings allowed researchers to propose that endogenous CTS might be considered as a novel class of steroid hormones. We focus our review on the analysis of the relative impact Na^+^_i_,K^+^_i_-mediated and -independent pathways in cellular responses evoked by CTS.

## 1. Introduction

**Figure 1:**
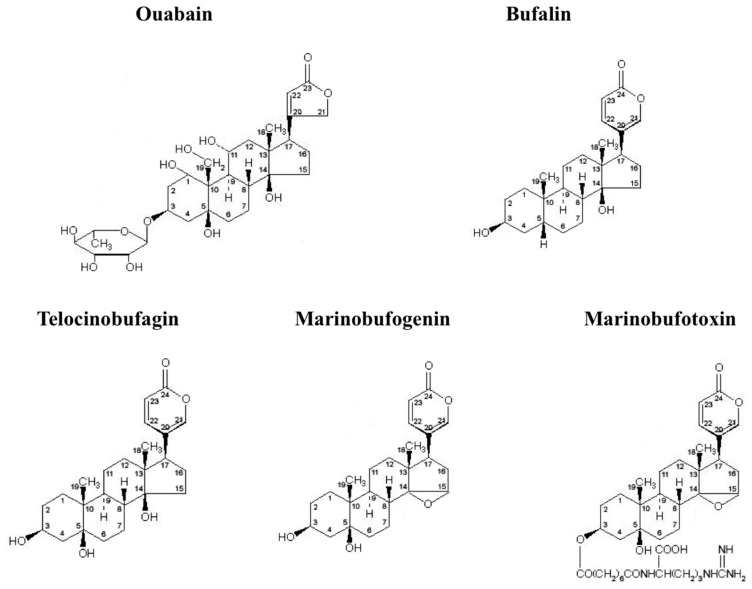
CTS identified in mammalian tissues.

Data on the beneficial effect of extracts from the leaves of *Digitalis purpurea* and *Digitalis lanata* in the treatment of heart failure published more that 200 years ago led to the isolation of digitoxin and digoxin, i.e., the first members of plant-derived cardiotonic steroids (CTS) known as cardenolides. Later on, other members of the CTS superfamily, bufadienolides, were isolated from amphibians. All of these compounds share a common structure formed by a steroid nucleus with a lactone ring at C-17 and a hydroxyl group at C-14. The five-membered and six-membered lactone rings are the most essential feature of cardenolides and bufadienolides, respectively (Figure 1). In 1938, Wood and Moe reported that treatment with cardenolides caused the accumulation of Na^+^ and loss of K^+^ in the canine ventricular musculature [1]. Fifteen years later, Schatzmann demonstrated that in human erythrocytes, these compounds inhibit energy-dependent accumulation of K^+^_o_ and extrusion of Na^+^_i_ [2]. Finally, two years after the discovery of Mg^2+^-dependent (Na^+^,K^+^)-stimulated adenosine triphosphatase (Na^+^,K^+^-ATPase) [3], Jens Skou reported that CTS completely suppressed the enzyme’s activity [4]. During the last two decades, several cardenolides and bufadienolides identified in mammals (Figure 1) were defined as endogenous CTS (for a review, see [5,6,7,8,9]).

**Figure 2:**
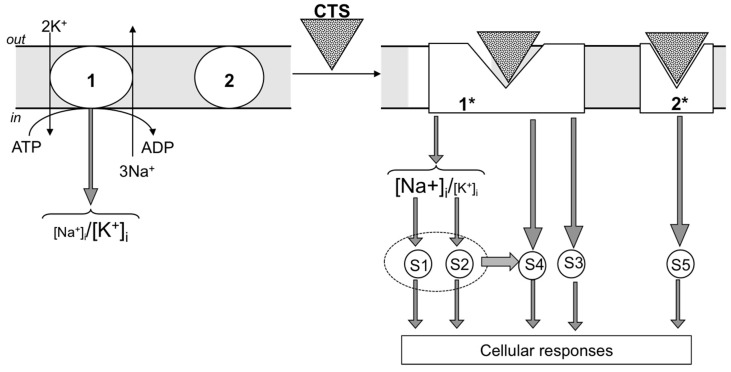
Intracellular signaling pathways triggered by CTS. 1, Na^+^,K^+^-ATPase; 2, CTS target(s) distinct from the Na^+^,K^+^-ATPase α-subunit; S1–S5, downstream signaling pathways. Different shapes of CTS targets (1 and 2) reflect their conformational transitions. For more details, see the text.

As predicted, exposure to CTS affects numerous cellular functions related to Na^+^,K^+^-ATPase activity and the maintenance of the transmembrane gradient of monovalent cations, such as electrical membrane potential (E_m_), cell volume, transepithelial movement of salt and osmotically-obliged water, Na^+^/H^+^ and Na^+^(K^+^)/Ca^2+^ exchange, symports of Na^+^ with inorganic phosphate, glucose, amino acids, nucleotides, etc. During the last two decades, it was shown that side-by-side with the above-listed cellular responses, CTS affect diverse non-canonical signaling pathways involved in the regulation of gene expression, membrane trafficking, cell adhesion, proliferation and death. Based on these findings, several research teams proposed that endogenous CTS might be considered as a novel class of steroid hormones [10,11,12,13,14]. Figure 2 shows that these cellular responses in CTS-treated cells might be mediated by unknown signaling pathways triggered by elevated [Na^+^]_i_ (pathway S1) or attenuated [K^+^]_i_ (pathway S2). These signals can be also evoked by conformational transition of the Na^+^,K^+^-ATPase that, in turn, triggers intracellular signals independently of the dissipation of transmembrane gradients of monovalent cations (pathway S3) or on the background of altered intracellular milieu caused by Na^+^,K^+^-ATPase inhibition and elevation of the [Na^+^]_i_/[K^+^] ratio (pathway S4). Finally, signals might be also generated by the interaction of CTS with targets distinct to the Na^+^,K^+^-ATPase (pathway S5). We focus our review on the analysis of the relative contribution of these signaling pathways in cellular responses triggered by CTS. Data on the physiological and pathophysiological implications of endogenous CTS obtained in experiments with anti-CTS antibodies and transgenic mice were out of the scope of our mini-review and subjected to detailed analysis elsewhere [8,9,13,15,16,17].

## 2. Na^+^,K^+^-ATPase as a CTS-Sensitive Ion Pump

Na^+^,K^+^-ATPase is an integral plasma membrane protein consisting of α- and β-subunits and detected in all types of animal cells. In accordance with the Albers–Post model, ATP hydrolysis by the larger α-subunit (~110 kD) leads to phosphorylation of the Asp369 residue that provides E_1_-E_2_ conformational transition and electrogenic ion transport (3Na^+^ vs. 2K^+^) at a baseline rate of 60–80 phosphorylation-dephosphorylation cycles per second. In addition to the ubiquitous α1-isoform, three other Na^+^,K^+^-ATPase α-subunits were detected by screening c-DNA libraries. These isoforms are expressed in a tissue-specific manner with high abundance in neuronal cells (α3 and α2), astrocytes and heart (α2), skeletal muscle (α3, α2), and testis (α4). Four isoforms of β-subunits encoding an ~35-kD protein have been demonstrated in mammals. All of them are highly glycosylated and are obligatory for the delivery, conformational stability and enzymatic activity. The 8-kD γ-subunit detected in highly-purified Na^+^,K^+^-ATPase from the kidney, as well as the other six members of the FXYD family sharing the Pro-Phe-X-Tyr-Asp motif also contribute to the enzyme activity regulation. For more details, see [15,18,19].

**Figure 3:**
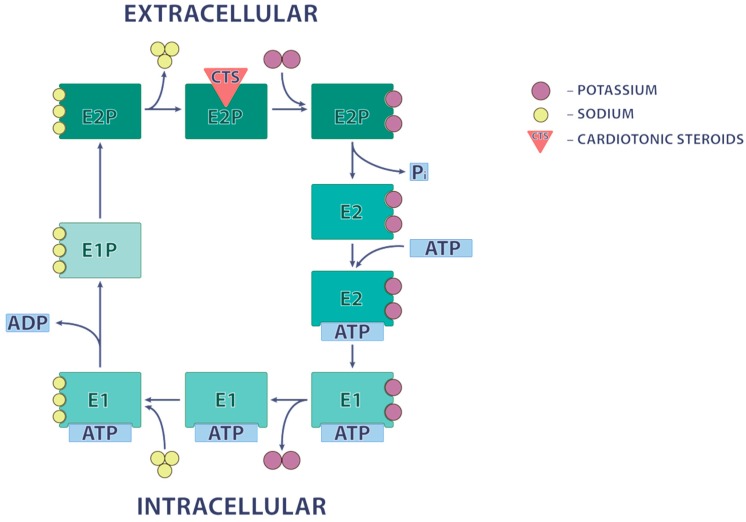
Catalytic cycle-dependent binding of CTS with the Na^+^,K^+^-ATPase α-subunit.

The mechanism of Na^+^,K^+^-ATPase inhibition by CTS was mainly explored with ouabain extracted from *Strophanthus gratus* and possessing much higher water solubility compared to other cardenolides and bufadienolides. The apparent affinity for ouabain is sharply increased in the absence of K^+^_o_ and in the presence of Na^+^_i_ [20], thus indicating that CTS specifically bind to the phosphorylated E_2_ state of Na^+^,K^+^-ATPase (Figure 3). Elegant studies performed by Lingrel and co-workers showed that at least 10 amino acid residues in transmembrane segments H1, H5 and H7, as well as in extracellular loops H1-H2, H5-H6 and H7-H8 α-subunit affect the affinity of Na^+^,K^+^-ATPase for ouabain [21]. Their crucial role in CTS binding was also confirmed by comparative analysis of Na^+^,K^+^-ATPase from different species. Thus, it was shown that the ~1000-fold decreased affinity for ouabain detected in the Na^+^,K^+^-ATPase α1-subunit form rat and mouse (CTS-resistant α1R-Na^+^,K^+^-ATPase) compared to other mammalian species (CTS-sensitive α1S-Na^+^,K^+^-ATPase) is caused by substitution of Gln111 and Asn122 by uncharged amino acids, such as Arg and Asp. The same amino acid substitution sharply decreased affinity for ouabain of α2- and α3-Na^+^,K^+^-ATPase [22]. Crystal structures of the Na^+^,K^+^-ATPase and of its cardiac glycoside complex have been identified [16,17].

## 3. Evidence for Na^+^_i_,K^+^_i_-Mediated Signaling

In this section, we briefly summarize the recent data on non-canonical CTS-induced cellular responses mediated by inhibition of the Na^+^,K^+^-ATPase and elevation of the [Na^+^]_i_/[K^+^]_i_ ratio. It should be noted that millemolar concentrations of CTS have been used in the part of experiments considered in this section. Keeping in mind that the endogenous CTS in mammals are mostly found at sub-nanomolar concentrations, the physiological significance of their actions detected in this range should be interpreted with caution.

### 3.1. Inhibition of Apoptosis

Almost 20 years ago, we surprisingly found that in rat vascular smooth muscle cells (VSMC) transfected with E1A-adenovirus (E1A-VSMC), ouabain sharply attenuated the development of apoptosis triggered by growth factor withdrawal, staurosporine and inhibitors of serine-threonine phosphatases [23]. The death of these cells was also suppressed by Na^+^,K^+^-ATPase inhibition in K^+^-free medium, whereas dissipation of the transmembrane gradient of monovalent cations in high-K^+^ medium completely abolished the anti-apoptotic action of ouabain [23]. Because the protection by ouabain was absent in K^+^-free, low-Na^+^ medium, we concluded that the anti-apoptotic signal was mediated by the gain of [Na^+^]_i_ rather than by the loss of [K^+^]_i_ [24,25] (Figure 2, pathway S1).

Additional experiments demonstrated that inhibitors of RNA and protein synthesis, such as actinomycin D and cycloheximide, abolished the protective effect of ouabain [26]. Deploying a rat multi-probe template set, we failed to detect differential expression of mRNA species encoding major pro- and anti-apoptotic proteins, such as Bcl-2, Bcl-xL, Bcl-xS, Bax and caspases-1–3, in ouabain-treated VSMC [27]. Keeping these negative data in mind, we adopted a proteomics approach to characterize a set of [Na^+^]_i_-sensitive genes. Several soluble proteins, including mortalin, whose expression is triggered by ouabain, were identified by mass spectrometry [28]. Northern and Western blotting confirmed the induction of mortalin expression in ouabain-treated VSMC and documented its mitochondrial localization. We established that, similarly to ouabain, transfection with mortalin delayed apoptosis in serum-deprived VSMC [28]. These experiments led us to the investigation of the mechanisms of the implications of the augmented [Na^+^]_i_/[K^+^]_i_ ratio in the regulation of gene transcription and translation considered in the next two sections.

### 3.2. Transcription

Three decades ago, it was demonstrated that exposure to ouabain of several cell types augments the expression of immediate response genes (IRG), such as c-Fos, c-Jun and Egr-1 [29,30,31,32,33]. Importantly, expression of c-Fos was noted at ouabain concentrations triggering inhibition of all cloned Na^+^,K^+^-ATPase, including rodent CTS-resistant α1R-Na^+^,K^+^-ATPase [34]. A key role of Na^+^,K^+^-ATPase inhibition in gene expression has been proven in our studies using VSMC from the rat aorta [35] and HeLa cells from the human kidney [36]. Side-by-side with ouabain, in both cell types, c-Fos expression was triggered by Na^+^,K^+^-ATPase inhibition in K^+^-depleted medium and correlated with the gain of [Na^+^] rather than the loss of [K^+^]_i._ These data demonstrated that CTS affect c-Fos expression via their interaction with Na^+^,K^+^-ATPase rather than other potential targets, and this signaling cascade is mediated by a rise in intracellular Na^+^ concentration (Figure 2, pathway S1).

Considering this finding, we identified ubiquitous and tissue-specific [Na^+^]_i_/[K^+^]_i_-sensitive transcriptomes by comparative analysis of differentially-expressed genes in VSMC from the rat aorta, HeLa cells and human umbilical vein endothelial cells (HUVEC) [37]. To augment [Na^+^]_i_ and reduce [K^+^]_i_, cells were treated for 3 h with ouabain or placed for the same time in the K^+^-free medium. Employing Affymetrix-based technology, we detected changes in expression levels of 684, 737 and 1839 transcripts in HeLa, HUVEC and VSMC, respectively, that were highly correlated between two treatments [37], thus demonstrating a key role of the Na^+^_i_/K^+^_i_-mediated mechanism of excitation-transcription coupling.

Riganti and co-workers suggested that prolonged incubation with CTS may affect gene expression via their interaction with steroid receptors distinct from the Na^+^,K^+^-ATPase α-subunit [13] (Figure 2, pathway S5). Indeed, it was shown that 24-h exposure of Caco-2 cells to 1 μM digoxin increased the content of multidrug resistance transporter MDR1 whose expression is controlled by a steroid xenobiotic receptor [38]. Smith and co-workers reported that 1 μM marinobufagenin sharply decreased the activity of the aldosterone-sensitive mineralocorticoid receptor [39]. Fujita-Sato et al. demonstrated that digoxin suppresses interleukin IL-17 production via its binding to the retinoic acid-related orphan nuclear receptor [40]. Keeping these data in mind, we compared dose-dependent actions of the long-term application of ouabain and marinobufagenin on gene expression and intracellular Na^+^ and K^+^ content [41]. The 96-h incubation of HUVEC with 3 nM ouabain or 30 nM marinobufagenin resulted in elevation of the [Na^+^]_i_/[K^+^]_i_ ratio by ~14- and 3-fold and differential expression of 880 and 484 transcripts, respectively. We failed to detect any differentially-expressed transcripts in 96-h incubation, with lower concentrations of ouabain and marinobufagenin having no action on intracellular content of monovalent cations. Thus, our results show that transcriptomic changes in CTS-treated HUVEC are triggered by elevation of the [Na^+^]_i_/[K^+^]_i_ ratio (Figure 2, pathway S1/S2), rather than by [Na^+^]_i_/[K^+^]_i_-independent signaling (pathway S3).

### 3.3. Translation

Since the initial observation of Lubin and Ennis [42], numerous laboratories demonstrated the requirement of K^+^ for protein synthesis, thus suggesting that side-by-side with transcription, CTS affect translation (for a review, see [43,44]). Indeed, in human fibroblasts, sustained Na^+^/K^+^-ATPase inhibition suppresses protein synthesis without any impact on mRNA function, ATP content and amino acid transport [45]. In reticulocytes, K^+^_i_ depletion inhibits the elongation step of globin synthesis without any impact on ribosome subunit assembly [46]. In these cells, elevation of [Na^+^]_i_ diminishes the efficiency of protein synthesis regulation by K^+^_i_, suggesting competition for the same binding site within a hypothetical K^+^_i_ sensor (Figure 2, pathway S2). As an alternative hypothesis, it might be proposed that elevation of [Na^+^]_i_ diminishes the transcription of elongation factors [47,48,49]. Indeed, we found that 6-h incubation of HUVEC with ouabain resulted in three-fold attenuation of mRNA encoding eukaryotic translation initiation factor 5 (eIF5) [41] that plays an ubiquitous role in protein synthesis by triggering GTP hydrolysis and mRNA translation [47,50].

It should be underlined that the effect of K^+^_i_ loss on protein synthesis is cell type specific. Thus, we did not see any significant effect on [^3^H]-leucine protein labelling after 24-h treatment of rat VSMC with ouabain [51]. Three hypotheses could explain these data. First, the K^+^_i_-sensitive element of the protein synthesis machinery is absent in VSMC. Second, K^+^_i_-insensitive transcription may be attributed to a specific class of mRNAs containing special elements in their promoters. Consistent with this hypothesis, Dever and co-workers reported that phosphorylation of the eukaryotic initiation factor 2 α-subunit (eIF2a) attenuates translation of mRNA with the exception of mRNA encoding activating transcription factor 4 (ATF4) and several other mRNAs with upstream open reading frames [52]. Third, attenuation of protein synthesis is masked by augmented transcription. Indeed, we discerned a six-fold elevation of total RNA synthesis in RASMC treated with ouabain for 10 h [51], which could be attributed to Na^+^_i_-mediated expression of c-Fos and other IRG considered in the previous section.

### 3.4. Tight Junctions and Cell Adhesion

Using chimerical constructs, it was shown that Na^+^,K^+^-ATPase contributes to cell motility, adhesion and tight junction formation due to the self-adhesive properties of the β-subunit (for a review, see [53,54,55]). Gupta and co-workers demonstrated that differences in dose-dependent attenuation of attachment by ouabain of human and monkey cells expressing CTS-sensitive α1S-Na^+^,K^+^-ATPase, vs. mouse and hamster cells, expressing CTS-resistant α1R-Na^+^,K^+^-ATPase, positively correlate with differences in dose-dependent inhibition of ^86^Rb influx [56]. At high concentrations, ouabain blocked tight junctions in Madin–Darby canine kidney (MDCK) [57], VSMC [58,59] and HeLa [58] cells and sharply attenuated the adhesion of COS-7 [60] and human retinal pigment epithelial cells [61]. Importantly, disruption of tight junction and adhesion in cells expressing α1R- and α1S-Na^+^,K^+^-ATPase was noted at a ouabain concentration ~1000 and 1 μM, respectively [57,58,59,60,62], i.e., in the range of full-scale inhibition of these enzyme. These actions of ouabain were abolished in Na^+^-free medium and were mimicked by Na^+^,K^+^-ATPase inhibition in K^+^-depleted medium [57,61,62,63]. These data strongly suggest that maintenance of transmembrane gradients of Na^+^ and K^+^ is obligatory to establish cell-to-cell communications and adhesion. The relative impact of the gain of Na^+^_i_ and the loss of K^+^_i_, as well as downstream intermediates of signal transduction remains a matter of speculation [64].

In contrast to the studies cited above, Larre and co-workers reported that three-day incubation of MDCK cells with ouabain at concentrations 10–50 nM does not disturb [K^+^]_i_, but increases the hermeticity of the tight junctions measured by transepithelial electrical and increases gap junctional communication between the cells [65,66] suggesting the implication of Na^+^_i_,K^+^_i_-independent signaling pathways [54,67]. Using pharmacological approaches, it was shown that these phenomena might be mediated by an ~2-fold elevation of c-Src and ERK1/2 MAPK phosphorylation [68]. Thus, additional experiments should be performed to examine the relative impact of Na^+^_i_,K^+^_i_-mediated and -independent signaling in the maintenance of tight junction and cell adhesion.

## 4. Evidence for Na^+^_i_,K^+^_i_-Independent Cellular Responses

### 4.1. Cell Proliferation

It was shown that at concentrations less than 10 nM, ouabain increased by 20–30% the proliferation of cultured canine and human VSMC [69,70], HUVEC [70,71], proximal tubule cells from opossum kidney [72] and human polycystic kidney cells [73] expressing α1S-Na^+^,K^+^-ATPase. At concentrations lower than 1000 nM, ouabain also augmented the growth of rat astrocytes [74], rat proximal tubule cells [70] and rat VSMC [70] expressing СTS-resistant α1R-Na^+^,K^+^-ATPase. In several investigations, it was shown that at these concentrations, ouabain does not inhibit Na^+^,K^+^-ATPase [69,71,72,73,75], suggesting the presence of a Na^+^_i_,K^+^_i_-independent mechanism of this phenomenon. It should be noted, however, that the lack of impact of low concentrations of ouabain documented in these studies might be due to the sharp differences of incubation times selected for the estimation of the proliferative effect of ouabain and its action on the Na^+^,K^+^-ATPase activity. Indeed, to study the proliferation, cells were incubated with ouabain for more than 24 h, whereas 15–30 min of incubation were used to assess the rate of ^86^Rb influx and ATPase activity [69,71,72,73,75,76]. This comment becomes important because of the slow actions of CTS at low concentrations on these parameters documented in human lymphocytes [77] and HUVEC [78]. Indeed, in 6 h, half-maximal elevation of [Na^+^]_i_ was detected at 100 nM ouabain, whereas in 24 and 48 h, the same increment was detected at ouabain concentrations of 3 and 10 nM, respectively [78]. Side-by-side with the slow kinetics of interaction of ouabain with α1-Na^+^,K^+^-ATPase observed in early studies [77], this phenomenon might be also caused by slow elevation of [Na^+^]_i_ and accumulation of Na^+^,K^+^-ATPase in the P-E_2_Na_3_ conformation possessing high affinity for CTS (Figure 3). The increment of [Na^+^]_i_ might be caused by the interaction of Na^+^,K^+^-ATPase with Na^+^/H^+^ exchange and activation of this carrier detected in renal epithelial cells treated with low concentrations of ouabain [79].

Keeping these comments in mind, we compared dose- and time-dependent actions of ouabain on the proliferation and intracellular Na^+^ and K^+^ content in HUVEC. We observed that 48–72-h exposure to low-dose ouabain increased cell growth of by 20–40%, whereas at concentrations higher than 30 nM, ouabain decreased cell proliferation [78]. Importantly, unlike high concentrations, prolonged exposure to 1 and 3 nM ouabain increased [K^+^]_i_ and decreased [Na^+^]_i_, resulting in attenuation of the [Na^+^]_i_/[K^+^]_i_ ratio by 30–50%. We also found that low concentrations of ouabain increased rather than decreased that rate of ^86^Rb influx, suggesting that elevation of the [Na^+^]_i_/[K^+^]_i_ ratio is caused by activation of the Na^+^,K^+^-ATPase. This conclusion is consistent with numerous studies demonstrating Na^+^,K^+^-ATPase activation by low concentrations of CTS. Thus, it was shown that ouabain at a concentration less than 10 nM decreases Na^+^_i_ in guinea pig atria [80,81] and augmented Na^+^/K^+^ pump-mediated ion current in single cardiac myocytes from guinea pig, dog and human hearts [82]. In human erythrocytes, activation of ^86^Rb uptake was observed at 0.1 nM ouabain [83], whereas in opossum and human kidney proximal tubule cells, augmented ^86^Rb uptake was seen at ouabain concentrations of 10 nM and 10 pM, respectively [72,79]. Activation by ouabain and other CTS was also documented in the study of ^86^Rb uptake in hippocampal slice cultures [84].

Based on the analysis of the kinetics of [^3^H]-ouabain binding, Ghysel-Burton and Godfraid proposed that activation and inhibition of the sodium pump by low and high concentrations of ouabain, respectively, is caused by its interaction with two distinct binding sites within the same Na^+^,K^+^-ATPase α-subunit [81]. However, the recent structural study failed to reveal the second CTS binding site within the α-subunit [85]. In contrast, Gao and co-workers suggested that the activatory action of ouabain is caused by its interaction with the α2- and α3-, but not with the α1-subunit [82]. It should be noted that the α1-Na^+^,K^+^-ATPase isoform is the only isoform detected in endothelial cells [86]. Moreover, we observed that low concentrations of ouabain activate purified α1-Na^+^,K^+^-ATPase from pig kidney [78]. In renal epithelial cells, activation of ^86^Rb uptake was caused by augmented delivery of α1-Na^+^,K^+^-ATPase to the basolateral membrane [79]. Keeping in mind the data on Na^+^,K^+^-ATPase functioning within the plasma membrane as α2β2-oligomer [87], it may be assumed that binding of low concentrations of CTS with α1β1 activates the enzyme, whereas occupation of α2β2 at higher CTS concentrations inhibits its activity. This hypothesis is currently being examined in our lab.

**Figure 4:**
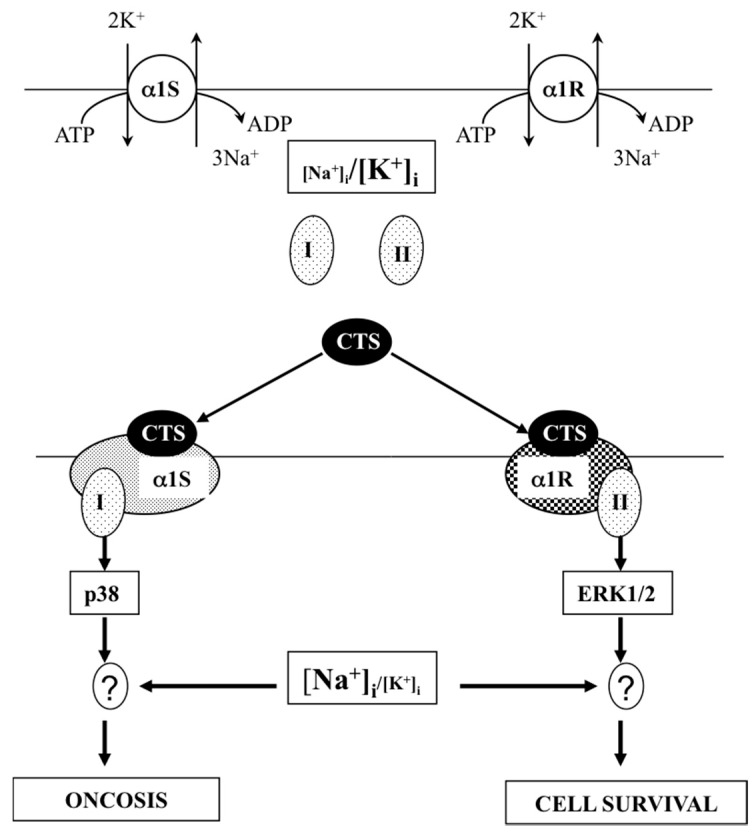
Hypothetical mechanisms underlying the distinct impact of cardiotonic steroids (CTS) on survival of cells, expressing the CTS-sensitive (α1S) and the CTS-resistant (α1R) Na^+^,K^+^-ATPase subunits. In both cases, saturating levels of CTS strongly increase the [Na^+^]_i_/[K^+^]_i_ ratio. In addition, CTS trigger distinct conformational changes in α1S and α1R isoforms that, in turn, affect their interactions with unknown protein partner(s) I and II. These subsequent signaling events lead to activation of p38 and ERK1/2 MAPK and result in cell death (oncosis) and survival, respectively. “?”, transcriptomic changes and/or other unknown steps of intracellular triggered by elevation of the [Na^+^]_i_/[K^+^]_i_ ratio. Modified from [92].

The data considered above strongly suggest that the activation of proliferation by low concentrations of CTS is by attenuation of [Na^+^]_i_ or/and elevation of [K^+^]_i_ (Figure 4). This hypothesis is consistent with early reports showing activation of this enzyme in cells treated with diverse proliferative stimuli. Thus, for example, increased Na^+^,K^+^-ATPase activity was documented in canine renal epithelial cells subjected to serum-derived growth factors [88] and in murine macrophages exposed to hemopoietic growth factors and interleukin-2 [89]. Activation of the Na^+^,K^+^-ATPase and elevation of intracellular K^+^ content was observed during the proliferation of human lymphocytes [90]. More, recently, Tian and co-workers demonstrated that si-RNA-mediated knockdown of α1-Na^+^,K^+^-ATPase decreased baseline proliferation of LLC-PK1 cells and abolished the increment of cell growth triggered by low concentrations of ouabain [91].

As shown above, sustained elevation of [Na^+^]_i_ increased the expression of hundreds of ubiquitous and cell type-specific genes via Ca^2+^_i_-mediated and -independent mechanisms of excitation-transcription coupling, whereas the loss of K^+^_i_ inhibits translation at the elongation step without any impact on ribosome subunit assembly. Recently, Ketchem et al. demonstrated that 15-min incubation of human kidney proximal cells with 10 pM resulted in an ~2-fold elevation of Na^+^,K^+^-ATPase-mediated ^86^Rb uptake and phosphorylation of EGFR, Src and ERK1/2. These effects were prevented by the angiotensin II type 1 receptor (AT1R) blocker candesartan. The authors concluded that in renal proximal tubule cells, ouabain stimulates Na^+^,K^+^-ATPase through an angiotensin/AT1R/Src/ERK1/2-dependent mechanism [93]. This signaling pathway is considered in more detail below. Its crosstalk with signals triggered by Na^+^,K^+^-ATPase activation and attenuation of the [Na^+^]_i_/[K^+^]_i_ ratio in the proliferative actions of low concentrations of CTS should be examined in forthcoming studies.

### 4.2. Membrane Trafficking

Using the pig renal proximal tubule cell line, LLC-PK1, Liu and co-workers reported that 12-h preincubation with 100 nM ouabain decreases ouabain-sensitive ^86^Rb uptake by 5–10-fold without any significant impact on total enzyme activity suggesting internalization of the Na^+^,K^+^-ATPase [94]. Later on, this conclusion was confirmed by the measurement of the content of membrane-bound Na^+^,K^+^-ATPase using the biotinylation assay [95]. Additional studies demonstrated that Na^+^,K^+^-ATPase internalization occurs via the canonical clathrin-dependent pathway of endocytosis mediated by activation of phosphotidylinositol-3 kinase (PI3K) and non-receptor tyrosine kinase Src (for a review, see [12]). It should be underlined that CTS-induced internalization is a cell type-specific phenomenon. Thus, preincubation with ouabain did not trigger Na^+^,K^+^-pump internalization in MDCK cells [94,96]. Moreover, in human and rat endothelial cells, as well as in rat astrocytes, ouabain decreased rather than increased endocytosis, measured as MTT uptake [97,98].

Yan and co-workers demonstrated that 1-h exposure of human renal HK-2 cells, LLC-PK1 and LLC-PK1 transfected with α1R-Na^+^,K^+^-ATPase to 0.01, 0.1 and 10 μM ouabain, respectively, triggered endocytosis of Na^+^,K^+^-ATPase, as well as the renal-specific isoform on Na^+^/H^+^ exchanger NHE3 [99]. Because 30-min exposure to the same concentrations of ouabain did not affect ^86^Rb influx, the authors assumed that internalization is mediated by Na^+^_i_,K^+^_i_-independent signaling pathways. Augmented endocytosis was also documented in human neuronal NT2 cells treated for 20 h with 20 nM bufalin. However, unlike LLC-PK1 cells, accumulation of vesicles within bufalin-treated NT2 cells was a result of inhibited recycling within the late endocytosis [100,101]. Importantly, 5-h exposure to 1 nM bufalin inhibited Na^+^,K^+^-pump by 2–3-fold [101]. Thus, additional experiments should be performed to estimate the relative contributions of Na^+^_i_,K^+^_i_-mediated and -independent signaling in cell type-specific actions of CTS on membrane trafficking.

### 4.3. Triggering of Oncosis

Numerous studies demonstrated tissue- and species-dependent actions of CTS on cell survival. Indeed, ouabain and other CTS, at concentrations that elicit full-scale inhibition of Na^+^,K^+^-ATPase and inversion of the [Na^+^]_i_/[K^+^]_i_ ratio, did not affect the survival of rat VSMC [23,51], Jurkat cells [102], NIH 3T3 mice fibroblasts [7], rat astrocytes [98] and rat aorta endothelial cells [92]. In contrast, prolonged exposure to ouabain evokes massive death of MDCK cells [103], porcine and human endothelial cells [24,104], as well as human astrocytes [92].

The death of ouabain-treated MDCK cells is represented by combined markers of “classic” necrosis (modest cell swelling, negligible labelling with nucleotides in the presence of terminal transferase, nuclei staining with cell-impermeable dyes, such as propidium iodide) and apoptosis (nuclear condensation seen in cells stained with cell-permeable dyes, such as Hoechst 33342, chromatin cleavage, caspase-3 activation) [103,105,106,107]). In accordance with cell volume behavior distinct from shrinkage seen in cells undergoing classic apoptosis, we termed the mode of СTS-induced cell death as “oncosis”, derived from the Greek word for swelling [25,27]. Surprisingly, unlike CTS, almost complete Na/K pump inhibition and full-scale increase of the [Na^+^]_i_/[K^+^]_i_ ratio evoked by K^+^-free medium did not affect the survival of MDCK cells [105,108].

As mentioned above, disruption of the tight junction and attenuation of cell adhesion were also noted at ouabain concentrations providing full-scale inhibition of the Na^+^,K^+^-pump. However, unlike oncosis, these actions of ouabain were abolished in Na^+^-free medium and were mimicked by Na^+^,K^+^-ATPase inhibition in K^+^-depleted medium [57,61,62,63,64]. These results show that the mechanisms of oncosis and the disruption of the tight junction by high concentrations of CTS are different.

Side-by-side with “classic” K^+^_o_-inhibited sites, bovine adrenocortical cells exhibit high-affinity ouabain-binding sites in the presence of 20 mM KCl, i.e., under conditions when its binding with the Na^+^,K^+^-ATPase is negligible [109]. Smith and co-workers reported that marinobufagenin interferes with the functions of mineralocorticoid receptors [39]. These data allowed us to propose that CTS trigger oncosis by interaction with targets distinct from the Na^+^,K^+^-ATPase α-subunit (Figure 2, pathway S5). To examine this hypothesis, we studied the dose-dependent effect of ouabain in K^+^-free medium. A similar left-hand shift was noted in the dose-dependent action of ouabain on Na^+^,K^+^ pump activity, as well as the death of MDCK and porcine endothelial cells incubated in K^+^-free compared to control K^+^-containing medium [24,105]. These data strongly suggest that CTS trigger oncosis via interaction with the Na^+^,K^+^-ATPase α-subunit rather than any other potential [K^+^]_o_-insensitive receptors. However, in contrast to the suppression of apoptosis in VSMC [23], the inhibition of Na^+^,K^+^-ATPase-mediated ion fluxes and elevation of the [Na^+^]_i_/[K^+^]_i_ ratio are not sufficient for triggering the cell death machinery.

The data considered above are consistent with two alternative mechanisms. First, oncosis occurs via Na^+^_i_,K^+^_i_-independent signaling triggered by interactions of CTS with the Na^+^,K^+^-ATPase (Figure 2, pathway S3). Second, propagation of the death signal triggered by interactions of CTS with the Na^+^,K^+^-ATPase occurs on the background of the elevated [Na^+^]_i_/[K^+^]_i_ ratio (Figure 2, pathway S4). To further examine these hypotheses, we transfected MDCK cells expressing α1S-Na^+^,K^+^-ATPase with rodent CTS-resistant α1R-Na^+^,K^+^-ATPase [110]. Six-hour treatment of α1R-cells with 1000 μM ouabain produced a similar increment of the [Na^+^]_i_/[K^+^]_i_ ratio detected in mock-transfected cells treated with 3 μM ouabain. However, in contrast to the massive death of mock-transfected cells exposed to 3 μM ouabain, α1R-cells survived after 24-h incubation with 1000 μM ouabain. Then, we compared dose-dependent actions of ouabain on intracellular Na^+^ and K^+^ content, cell survival and mitogen-activated protein kinases (MAPK) in human and rat vascular smooth muscle cells (HASMC and RASMC) and human and rat endothelial cells (HUVEC and RAEC) [92]. Six-hour exposure of HASMC and HUVEC to 3 μM ouabain dramatically increased the intracellular [Na^+^]/[K^+^] ratio to the same extent as in RASMC and RAEC treated with 3000 μM ouabain. In contrast to human cells, we did not detect any effect of the 3000–5000 μM ouabain on the survival of rat cells, as well as smooth muscle cells from mouse aorta (MASMC). In HUVEC, ouabain led to phosphorylation of p38 MAPK, whereas in RAEC, it stimulated phosphorylation of ERK1/2. Importantly, unlike the wild-type α1^R/R^ mouse, ouabain triggered death of smooth muscle cells from α1^S/S^ mouse expressing human α1S-Na^+^,K^+^-ATPase [92].

Overall, our results demonstrate that the drastic differences in cytotoxic action of ouabain on human and rodent cells are caused by unique features of α1S/α1R-Na^+^,K^+^-ATPase, rather than by any downstream CTS-sensitive/-resistant components of the cell death machinery. They also suggest that elevation of the [Na^+^]_i_/[K^+^]_i_ ratio contributes to the transduction of death signaling triggered by the interaction of CTS with α1S-Na^+^,K^+^-ATPase. We proposed that CTS trigger distinct conformation transitions of α1S- and α1R-Na^+^,K^+^-ATPase, resulting in their interaction with hypothetical adaptor proteins I and II, respectively (Figure 4). In the case of rodent cells, ouabain is not toxic, possibly because the signaling cascade triggered by its interaction with α1R-Na^+^,K^+^-ATPase leads to cytoprotective activation of ERK1/2. This is in contrast to activation of p38 MAPK seen in ouabain-treated HUVEC [92] and MDCK cells [111] expressing α1S-Na^+^,K^+^-ATPase. Sustained elevation of the [Na^+^]_i_/[K^+^]_i_ ratio is obligatory for the signal transduction (Figure 2, pathway S4) via differential expression of hundreds of ubiquitous and cell-type specific genes, including potent regulators of cell differentiation, proliferation and death [37].

One critical implication of the present findings is related to development of anti-cancer therapies based on CTS. Epidemiological observations identified decreased occurrence of leukemia, as well as breast, prostate and lung cancer in the patients with heart failure, who were treated with *Digitalis* [112,113,114]. Therefore, numerous studies screened for the novel anticancer CTS compounds, using rodents injected with human malignant cells (for a review, see [115,116,117]). The data considered above show that such an approach can be highly problematic since low concentrations of CTS may trigger undesirable cell death in human, but not rodent tissues.

### 4.4. Different CTS Trigger Distinct Cellular Responses

Another piece of evidence for Na^+^_i_,K^+^_i_-independent signaling is based on data showing that different CTS evoke distinct in vitro and in vivo responses. Thus, for example, bufalin, but not ouabain evoked differentiation of human leukemia cells, whereas marinobufagenin, but not ouabain and digoxin increased the constriction of uterine vessels [13,118]. We observed that unlike ouabain, the complete Na^+^,K^+^-ATPase inhibition by marinobufagenin and marinobufotoxin does not trigger oncosis of MDCK cells [119]. In contrast to ouabain, chronic treatment with digoxin did not raise blood pressure and even diminished the hypertensive action of ouabain in rats [120,121,122]. Administration of antibodies against marinobufagenin, but not ouabain, lowered blood pressure in rats with salt-sensitive hypertension [123,124] and chronic renal failure [125]. Administration of ouabain delayed the development of digoxin-induced arrhythmia and cardiac fibrillation in anaesthetized guinea pigs [126]. Zulian and co-workers found that in vivo administration of ouabain, but not digoxin, leads to augmented phosphorylation of Src and attenuated phosphorylation of ERK1/2. They also found that digoxin completely abolished the increment of the expression of Na^+^/Ca^2+^ exchanger NCX1 and receptor operated channel TRPC6 triggered by 72-h exposure of mesenteric artery smooth muscle cells to 100 nM ouabain [127].

**Figure 5:**
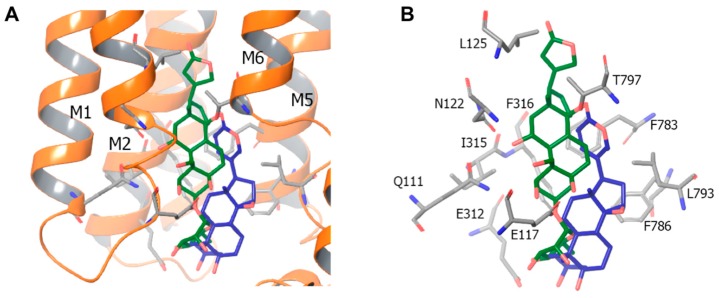
Location of ouabain (green) and marinobufagenin (blue) in the structure of Na^+^,K^+^-ATPase. (**A**) M1, M2, M5 and M6 are transmembrane segments of the Na^+^,K^+^-ATPase α1-subunit contributing to CTS binding; (**B**) amino acid residues of the Na^+^,K^+^-ATPase α1-subunit involved in the complex formation are indicated. The structural elements of the Na^+^,K^+^-ATPase in complexes with ouabain and marinobufagenin are shown in grey and pink, respectively. Modified from [129].

Assuming that Na^+^,K^+^-ATPase is the only receptor for CTS, the possible mechanism of their diversity may lie in (i) their different affinity for different Na^+^,K^+^-ATPase isoform or/and (ii) different conformation transition of α-Na^+^,K^+^-ATPase triggered by distinct CTS that, in turn, triggers diverse downstream signals. Karlish and co-workers examined the first hypothesis utilizing the α1β1-, α2β1 and α3β1-isoform of human Na^+^,K^+^-ATPase expressed in yeast [128]. They demonstrated that apparent affinity of these isoforms for cardiac glycosides differs by less than three-fold, whereas their affinity for marinobufagenin is decreased compared to ouabain by 100–200-fold. More recently, this research team synthesized CTS derivatives whose selectivity for α2- vs. α1-isoform is increased by seven-fold [129,130]. The second hypothesis is consistent with our recent data obtained by isothermal titration microcalorimetry of purified α1β1-isozyme from duck salt glands [131]. These data indicate that the locations of ouabain and marinobufagenin within the Na^+^,K^+^-ATPase a1-subunit are different: ouabain is completely submersed in the split formed by transmembrane segments M1, M2, M5 and M6, whereas marinobufagenin only partially penetrates within this area of the enzyme (Figure 5). Using the same experimental approach, we found that unlike ouabain, E1-E2P conformational transition does not significantly affect the apparent affinity of the purified Na^+^,K^+^-ATPase for marinobufagenin [131]. Viewed collectively, these data indicate different actions of ouabain and marinobufagenin on Na^+^,K^+^-ATPase conformation that, in turn, may leads to the activation of different signaling and cellular responses.

## 5. Search for Intracellular Na^+^ and K^+^ Sensors

According to the generally accepted paradigm, Na^+^_i_/K^+^_i_-sensitive mechanism of excitation-transcription coupling might be driven by elevation of [Ca^2+^]_i_ and activation of several Ca^2+^-sensitive pathways, a phenomenon termed excitation-transcription coupling [132,133,134]. Numerous research teams reported that dissipation of the transmembrane gradient of monovalent cations typically leads to increases in [Ca^2+^]_i_ via activation of the Na^+^/Ca^2+^ exchanger and/or voltage-gated Ca^2+^ channels. Elevation of [Ca^2+^]_i_, in turn, leads to its interaction with calmodulin and other Ca^2+^_i_ sensors and activation of Ca^2+^ response elements detected within promoters in hundreds of genes (for a comprehensive review, see [135,136,137]). Thus, for example, sharp elevation of cyclooxygenase 2 (COX-2) mRNA content seen in ouabain-treated human lung fibroblasts was completely abolished by Na^+^/Ca^2+^ exchanger inhibitor KB-R4943 [138].

In early studies, we found that 2-h exposure of rat VSMC to saturated concentrations of ouabain resulted in a 10- and four-fold increment of c-Fos and c-Jun [35]. Because the c-Fos promoter contains the Ca^2+^+cAMP response element (CRE) [136,139], its augmented expression in ouabain-treated cells might be mediated by the [Ca^2+^]_i_ increment caused by depolarization and opening of voltage-gated Ca^2+^ channels. Indeed, VSMC depolarization in high-K^+^ medium leads to activation of c-Fos expression as observed in ouabain-treated cells. However, unlike high-K^+^ medium, c-Fos expression in ouabain-treated cells is not affected by inhibition of L-type Ca^2+^ channels with nicardipine. Moreover, we observed that augmented c-Fos expression evoked by ouabain was preserved in Ca^2+^-free medium and in the presence of extracellular (EGTA) and intracellular (BAPTA) Ca^2+^ chelators [35]. Importantly, unlike rat VSMC, the increment of cFos expression triggered by 45-min exposure of neonatal rat cardiac myocytes to 100 μM ouabain was completely abolished by EGTA and BAPTA [30], suggesting the cell type-specific mechanism of Ca^2+^_i_-mediated signaling.

In the next experiments, we examine transcriptomic changes in rat VSMC, HeLa and HUVEC treated with ouabain in Ca^2+^-free media supplemented with extracellular and intracellular Ca^2+^ chelators. Surprisingly, this procedure elevated rather than decreased the number of ubiquitous and cell-type-specific Na^+^_i_/K^+^_i_-sensitive genes [37]. Among the ubiquitous Na^+^_i_/K^+^_i_-sensitive genes whose expression was regulated independently of the presence of Ca^2+^ chelators by more than three-fold, we discovered several transcription factors (*Fos*, *Jun*, *Hes1*, *Nfkbia*), interleukin-6, the protein phosphatase 1 regulatory subunit, dual specificity phosphatase (*Dusp8*), *Cox-2* and cyclin L1, whereas the expression of metallopeptidase *Adamts1*, adrenomedullin, *Dups1*, *Dusp10* and *Dusp16* was detected exclusively in Ca^2+^-depleted cells. These data allowed us to conclude that both canonical Ca^2+^_i_-mediated and novel Ca^2+^_i_-independent mechanisms contribute to transcriptomic changes evoked by the elevation of the [Na^+^]_i_/[K^+^]_i_ ratio in CTS-treated cells.

To further explore the role of Ca^2+^ in the expression of Na^+^_i_/K^+^_i_-sensitive genes, we compared transcriptomes of VSMC subjected to Na^+^,K^+^-ATPase inhibition and treated with extra- and intra-cellular Ca^2+^ chelators [140]. We found a highly significant (*p* < 10^−12^) positive (*R*^2^ > 0.51) correlation between levels of expression of 2071 transcripts whose expression was affected by both stimuli. Among genes whose expression in Ca^2+^-depleted cells was augmented by more than seven-fold, we noted cyclic AMP-dependent transcription factor *Atf3*, early growth response protein *Egr1* and nuclear receptor subfamily 4, group A member *Nr4a1*. Importantly, Ca^2+^ depletion resulted in elevation of [Na^+^]_i_ and attenuation of [K^+^]_i_ by ~3- and two-fold, respectively. Consistent with previous reports [141,142,143], the elevated [Na^+^]_i_/[K^+^]_i_ ratio seen in Ca^2+^-depleted cells was caused by augmented permeability of the plasma membrane for monovalent cations triggered by the presence of extracellular Ca^2+^ chelator EGTA [140]. Thus, novel experimental approaches should be developed to examine the relative impact of Ca^2+^-mediated- and -independent signaling in overall transcriptomic changes triggered by the augmented [Na^+^]_i_/[K^+^]_i_ ratio.

**Figure 6:**
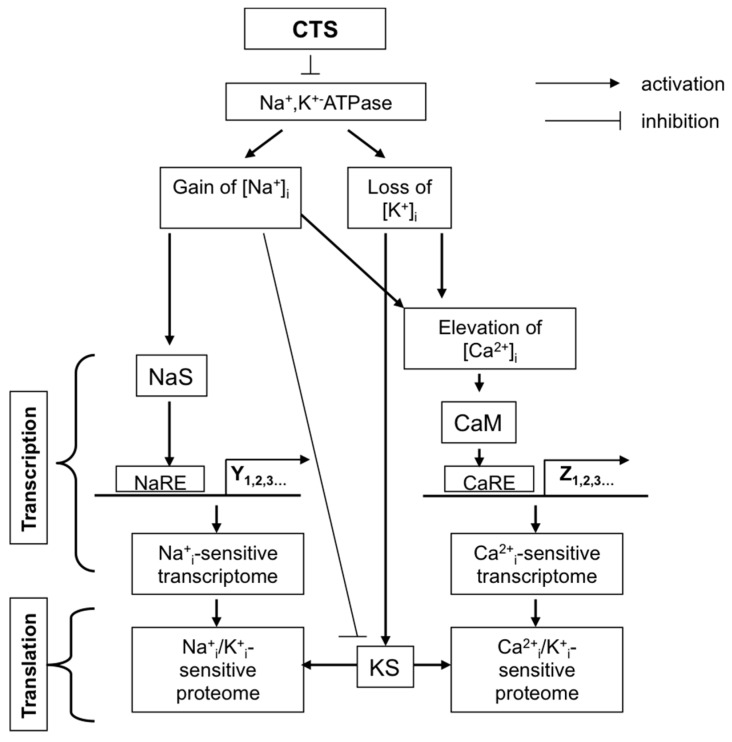
Possible mechanisms of the implication of intracellular Na^+^, K^+^ and Ca^2+^ in excitation-transcription and excitation-translation coupling. CaM, calmodulin and other intracellular Ca^2+^ sensors; CaRE, Ca^2+^-sensitive response elements; KS and NaS, intracellular K^+^ and Na^+^ sensors, respectively; NaRE, Na^+^-sensitive response elements. For more details, see the text.

It is generally accepted that transcription is under the control of proteins interacting with specific response elements within 5′- and 3′-untranslated region (UTR). Thus, for example, *c-Fos* 5′-UTR contains serum response element (SRE) and Ca^2+^+cAMP response element (CRE) activated by [Ca^2+^]_i_ increments in the cytoplasm and nucleus, respectively [144]. We proposed that similar to Ca^2+^_i_-mediated-signaling elevation of [Na^+^]_i_ may affect gene expression via the interaction of an unknown Na^+^_i_-sensor with a hypothetical Na^+^ response element (NaRE) located within promoters of c-Fos and other ubiquitous [Na^+^]_i_/[K^+^]_i_-sensitive genes (Figure 6). Positive results with this approach could identify NaRE binding protein (NaREBP) by mating yeast transformed with NaRE of the c-Fos 5′-UTR. However, with the construct containing CRE and all other known transcription elements of the *c-Fos* promoter, we failed to detect any significant elevation of luciferase expression in HeLa cells subjected to 6-h inhibition of Na^+^/K^+^-ATPase that contrasted with massive accumulation of endogenous c-Fos mRNA and immunoreactive protein in ouabain-treated HeLa cells [36].

Several hypotheses could be proposed to explain negative results obtained in this study: (i) NaRE is located within introns or/and the c-Fos 3′-UTR; (ii) the [Na^+^]_i_/[K^+^]_i_ ratio elevation affects gene expression via epigenetic modification of the DNA, histones or nucleosome remodeling, i.e., regulatory mechanism having a major impact on diverse cellular functions [144]; importantly, the epigenetic mechanism of gene expression does not contribute to the regulation of L-luc transcription in the plasmid employed in our experiments [36]; (iii) increasing evidence indicates that gene activation or silencing is under the complex control of three-dimensional (3D) positioning of genetic materials and chromatin in the nuclear space (for review, see [145]). It may be proposed that the augmented [Na^+^]_i_/[K^+^]_i_ ratio affects gene transcription by changing the 3D organization of the DNA-chromatin complex. These hypotheses should be verified in forthcoming studies.

## 6. Search for Na^+^_i_,K^+^_i_-Independent Signaling Pathways

It is well documented that the Na^+^,K^+^-ATPase α-subunit interacts with dozens of proteins involved in the regulation of intracellular retention (Akt kinase substrate AS160), clustering within the plasma membrane (caveolin, adducing, cofilin), proteasomal degradation (β-subunit of the coating protein COP-1), Na^+^_o_-sensitive Na^+^ channel Na_x_, salt-inducible kinase SIK1, etc. (for a review, see [146]). Data considered above show that elevation of the [Na^+^]_i_/K^+^]_i_ ratio per se is not sufficient to explain oncosis triggered by long-term exposure of cells expressing α1S-Na^+^,K^+^-ATPase to high concentrations of CTS. Xie and Askari were probably the first to propose an implication of Na^+^_i_,K^+^_i_-independent signals in the regulation of cell function by CTS [10]. Studies examining this hypothesis are briefly considered below.

### 6.1. Src-Kinase-Mediated Signaling

**Figure 7:**
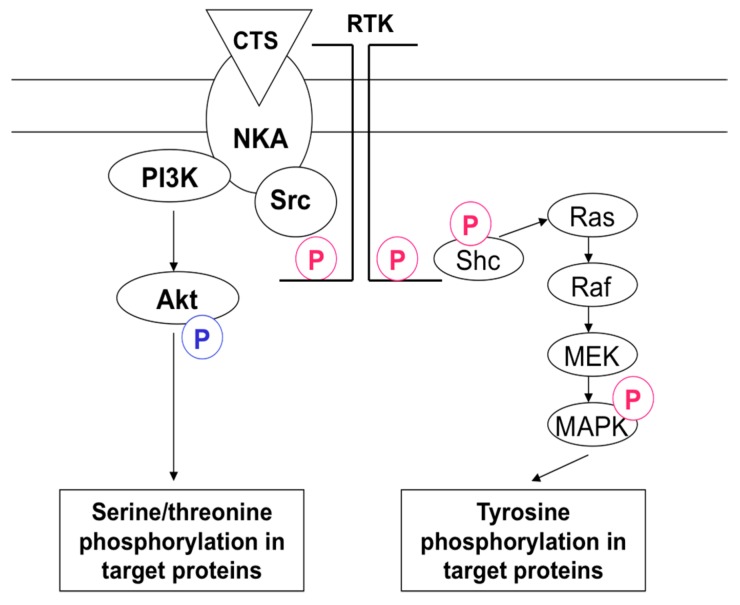
Signaling pathways involved in activation of Akt and MAPK by CTS. NKA, Na^+^,K^+^-ATPase; RTK, receptor tyrosine kinases. Phosphorylation of serine/threonine and tyrosine residues is shown in blue and red, respectively. For more details, see the text.

In a series of elegant studies, Xie and co-workers documented that ouabain evokes the activation of the membrane-associated non-receptor tyrosine kinase Src. Activated Src leads to phosphorylation of epidermal growth factor receptor (EGFR) and other receptor tyrosine kinases (RTK) that, in turn, activate diverse downstream intermediates of the signaling cascade, including phospholipase C (PLC-γ), G-protein Ras and mitogen-activated protein kinases (MAPK) [12] (Figure 7). The first evidence supporting this signaling pathway came from data showing dose- and time-dependent tyrosine phosphorylation in several types of CTS-treated cells. Thus, in cardiac myocytes, A7r5, HeLa and L929 cells’ exposure to ouabain resulted in rapid activation of Src, its interaction with EGFR and tyrosine phosphorylation of several proteins that was abolished in the presence of Src kinase inhibitors (PP2 and herbimycin A) and inhibitor of RTK AG1478 [147,148]. Importantly, using transfected pig renal epithelial cells (PY-17), it was shown that ouabain activates Src and ERK MAPK in cells expressing α1-, but not α2-Na^+^,K^+^-ATPase [149].

Later on Liang and co-workers demonstrated that the rupture of caveolae by cholesterol or caveolin-1 depletion resulted in attenuation of Src-mediated signaling triggered by ouabain and activation of ouabain-sensitive ^86^Rb influx, suggesting the presence within caveolae of non-pumping pools of the Na^+^,K^+^-ATPase involved in the triggering of Na^+^_i_,K^+^_i_-independent signals [150]. Detailed mapping of the α1-Na^+^,K^+^-ATPase nucleotide binding domain led to the identification of a 20-amino acid peptide (Ser-415 to Gln-434, NaKtide) that inhibits Scr with IC_50_ of 70 nM. The positively-charged NaKtide derivatives penetrated LLC-PK1 cells and blocked ouabain-induced activation of Src and ERK MAPK [151]. The same research team transfected α1-Na^+^,K^+^-ATPase knockdown PY-17 cells with expression vectors of wild-type enzyme and α1 carrying mutations in the NaKtide region. They selected mutants restoring ouabain-sensitive Na^+^,K^+^-ATPase and found that A420P and A425P mutants were incapable of interacting with Src and provide ouabain-dependent regulation of Src activity [152]. More recently, the same research team reported that the single Pro224 mutation within rat α1-Na^+^,K^+^-ATPase inhibits Src-mediated signaling pathway without any impact on the dose-dependent inhibition by ouabain of Na^+^,K^+^-ATPase activity and the rate of ^86^Rb uptake [153].

Since the initial observation, it has been proposed that the signaling cascade triggered by the interaction of the Na^+^,K^+^-ATPase with Src is independent of any changes in intracellular Na^+^, K^+^ and Ca^2+^ concentrations [10,12]. Indeed, initial publications reported augmented tyrosine phosphorylation of EGFR and several other proteins at ouabain concentrations having no significant action on ^86^Rb influx and intracellular Na^+^ content [69,75,154]. Recently, it was shown that in the murine spermatogenic cell line GC-2 abundant with α4-Na^+^,K^+^-ATPase, an ~2-fold elevation of phosphorylation of ERK1/2, as well as transcription factors GREB and ATF-1 was detected in 30-min exposure to 10^−11^ M ouabain, suggesting Na^+^_I_,K^+^-independent signaling [155]. Unlike α4-Na^+^,K^+^-ATPase, CTS do not affect Src activity in α1-Na^+^,K^+^-ATPase knockdown pig kidney cells PY-17 expressing the α3-isoform of this enzyme [156]. Using FRET technology, Tian et al. found that in LLC-PK1 cells, ouabain triggers dissociation of the Src kinase domain from the α1-Na^+^,K^+^-ATPase nucleotide binding domain resulting in tyrosine phosphorylation and activation of this enzyme [157]. More recently, however, Gable et al. reported that attenuation of Src phosphorylation by ouabain seen in cell-free systems is primary due to the ATP-sparing effect and cannot be considered as evidence for CTS-induced interaction of Na^+^,K^+^-ATPase and Src [158].

In contrast to the above-cited studies, several research teams reported that CTS trigger Src-mediated signaling at concentrations inhibiting the Na^+^,K^+^-ATPase [148,157,159,160,161,162]. Moreover, the augmented tyrosine phosphorylation seen in CTS-treated cells was mimicked by Na^+^,K^+^-ATPase inhibition in K^+^-depleted medium [147]. Later on, using purified Na^+^,K^+^-ATPase, it was shown that its interaction with Src measured by Src phosphorylation is suppressed by elevation of K^+^ and attenuation of Na^+^. These data indicated that side-by-side with CTS, any other stimuli triggering the E_1_ to E_2_ conformational transition are sufficient to release the kinase domain and activate the associated Src [163]. Indeed, CTS was more effective in inhibiting Src activity in the I279A mutant of α1-Na^+^,K^+^-ATPAse keeping the pump in E_1_ state than either wild-type or the F286A mutant with increased E_2_ state [164]. Viewed collectively, these data strongly suggest that at least in several cell types, an elevated [Na^+^]_i_/K^+^]_i_ ratio contributes to the triggering/progression of Src-mediated signaling in CTS-treated cells (Figure 2, pathway S4).

### 6.2. PI3K-Akt-Mediated Signaling

Liu et al. reported that in cultured neonatal rat cardiac myocytes, 50 μM ouabain activates protein kinase B, also known as Akt, i.e., a serine/threonine-specific protein kinase that plays a key role in multiple cellular processes including cell proliferation, apoptosis, transcription and cell migration. They also found that ouabain causes a transient increase of phosphatidylinositol-3,4,5-triphosphate (PIP3) content and leads to co-immunoprecipitation of the p85 subunit of class IA PI3K and Na^+^,K^+^-ATPase α-subunit that was abolished in the presence of phosphatidylinositol 3-kinase (PI3K) inhibitors [165]. To examine the role of Src in the triggering of PI3K/Akt signaling, Wu and co-workers employed mouse fibroblasts lacing Src (SYF cells) and control Src^+++^ cells. They found that ouabain triggers accumulation of PIP3, activation of Akt and PI3K1A, as well as co-immunoprecipitation of the p85 subunit of PI3KIA and Na^+^,K^+^-ATPase in both types of cells and was insensitive to the presence of Src inhibitor PP2. These results allowed hypothesizing that activation of Akt is triggered by CTS-induced interaction of a proline-rich domain of the α-subunit of Na^+^,K^+^-ATPase with the SH3 domain of the p85 subunit of PI3KIA (Figure 7) [161].

Similar to Src-mediated signaling, activation of PI3K and Akt was significantly reduced in cardiomyocytes isolated from caveolin-1 knockout mice, suggesting that this signaling pathway mainly occurs within caveolae [166]. Wu and co-workers reported that ouabain did not induce PIP2 accumulation and Akt activation in cardiomyocytes isolated from transgenic mouse deficient in p85 PI3KIA (p85-KO) [167]. Moreover, early ouabain treatment attenuated cardiac hypertrophy and fibrosis in mice subjected to transverse aortic constriction. Importantly, the action of ouabain was absent in p85-KO mice, thus suggesting that side-by-side with the positive inotropic effect, the treatment with *Digitalis* prevents pathological cardiac hypertrophy and heart failure through activation of PI3KIA [167]. Our recent study demonstrated that anti-fibrotic action of cardiac glycosides documented by the suppression of myofibroblast differentiation in TGF-β-treated human lung fibroblasts is mediated by dissipation of transmembrane gradients of monovalent cations [138,168]. To the best of our knowledge, the role of Na^+^,K^+^-ATPase inhibition and the elevated [Na^+^]_i_/K^+^]_i_ ratio in PI3K/Akt-mediated signaling triggered by CTS has not been explored yet.

### 6.3. Ca^2+^_i_-Oscillations

In 2001, Aperia and co-workers reported that the addition of 50–250 μM ouabain causing partial Na^+^,K^+^-ATPase inhibition increased the amplitude of low-frequency [Ca^2+^]_i_ oscillation in rat proximal tubule cells, which were abolished in Ca^2+^-free medium and by L-type Ca^2+^ channel blocker nifedipine [169]. Later on, [Ca^2+^]_i_ oscillations were detected in human aortic endothelial cells exposed to low concentrations of ouabain [71]. It is well-documented that [Ca^2+^]_i_ oscillations trigger the frequency-specific activation of transcription factors [170,171]. Indeed, blockage of [Ca^2+^]_i_ oscillations abolished ouabain-induced activation of NF-kB and CREB documented by their transportation into the nucleus and phosphorylation, respectively [76,169]. In human COS-7 cells [Ca^2+^]_i_, these oscillations were found in the presence of 100 nM ouabain causing a 10% inhibition of the rate of ^86^Rb influx [71,172]. The same oscillations were also detected in the presence of 100 nM digoxin and marinobufagenin [173].

**Figure 8:**
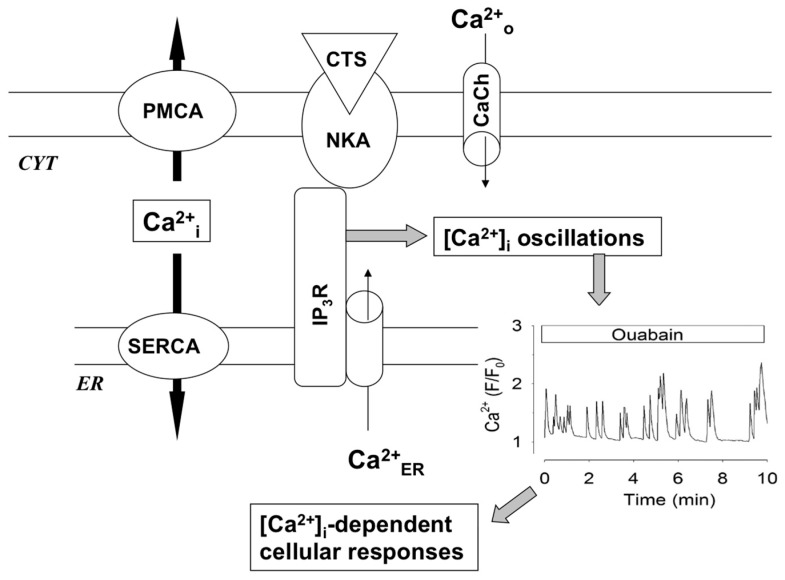
Ca^2+^_i_ oscillations triggered by CTS via the interaction of the Na^+^,K^+^-ATPase (NKA) with the inositol 1,4,5-triphosphate receptor (IP_3_R). Side-by-side with IP3R, oscillations of [Ca^2+^]_i_ are under the control of channels providing Ca^2+^ influx (CaCh) and Ca^2+^-ATPases localized in the plasma membrane (PMCA) and endoplasmic reticulum (SERCA). Representative Ca^2+^_i_ oscillations documented in 6 h of exposure of rat cortical neurons to 1 μM ouabain [74] are shown within the insert. CYT, cytoplasm; ER, endoplasmic reticulum.

Numerous studies demonstrated that [Ca^2+^]_i_ oscillations occur as an interplay between plasma membrane channels providing Ca^2+^ influx, Ca^2+^ pumps of the plasma membrane and endoplasmic reticulum, as well as Ca^2+^ release from endoplasmic reticulum triggered by activation of ryanodine or inositol-1,4,5-triphosphate receptors (InsP_3_R) (for a review, see [174]). Miyakawa-Naito and co-workers reported that [Ca^2+^]_i_ oscillations seen in ouabain-treated cells are caused by conformation transition of the Na^+^,K^+^-ATPase α-subunit and its interaction with InsP_3_R (Figure 8). Indeed, activation of the IP3R was independent of the activation of phospholipase C and the release of inositol 1,4,5-trisphosphate [175]. This mechanism has been also confirmed by the fluorescence resonance energy transfer (FRET) technique, showing the presence of the Na^+^,K^+^-ATPase-InsP_3_R signaling microdomain [175], by identification of the InsP_3_R-binding motif in the N-terminus of Na^+^,K^+^-ATPase α-subunits [172] and by inhibition of ouabain-induced [Ca^2+^]_i_ oscillations in cells overexpressing a peptide corresponding to the InsP_3_R-binding fragment of the Na^+^,K^+^-ATPase N-terminus [172]. Ankyrin by binding with N-terminus of Na^+^,K^+^-ATPase α-subunit and InsP_3_R acts as a stabilizing scaffolding protein providing the implication of the cytoskeleton network in the regulation of this signaling pathway [176].

Importantly, unlike modest ouabain concentrations, full Na^+^,K^+^-ATPase inhibition with 2 mM ouabain did not cause [Ca^2+^]_i_ oscillations, but led to sustained increase in [Ca^2+^]_i_. It was also shown that attenuation of [K^+^]_o_ from 4.0 down to 0.5 mM resulted in the same elevation of [Na^+^]_i_ as in the presence of 250 μM ouabain. However, in contrast to ouabain, lowering [K^+^]_o_ abolished rather than increased [Ca^2+^]_i_ oscillation [169]. Based on these observation, the authors proposed that [Ca^2+^]_i_ oscillations seen in ouabain-treated cells are not a primary result of Na^+^,K^+^-ATPase inhibition. It should be noted, however, that lowering [K^+^]_o_ may inhibit the L-type Ca^2+^ channel via plasma membrane hyperpolarization, thus contributing to regulation of [Ca^2+^]_i_ oscillations independently of partial Na^+^,K^+^-ATPase inhibition. To test the role of E_m_ in [Ca^2+^]_i_ oscillations, Desfrere and co-workers employed rat cerebral cortical neurons. In these cells, [Ca^2+^]_i_ oscillations were detected in the presence of 1 μM ouabain. At this concentration, ouabain inhibited ^86^Rb influx by 25% and did not affect E_m_ estimated by whole cell patch-clamp recording [76]. It should be mentioned that E_m_ was recording during 2–5 min, whereas [Ca^2+^]_i_ oscillations were seen in 6 h of ouabain addition. Thus, additional approaches should be developed to examine the role of the dissipation of the transmembrane gradient of monovalent cations and altered electrical membrane potential in [Ca^2+^]_i_ oscillation evoked by CTS-induced interaction of the Na^+^,K^+^-ATPase with the InsP_3_ receptor.

## 7. Conclusions and Unresolved Issues

**Figure 9:**
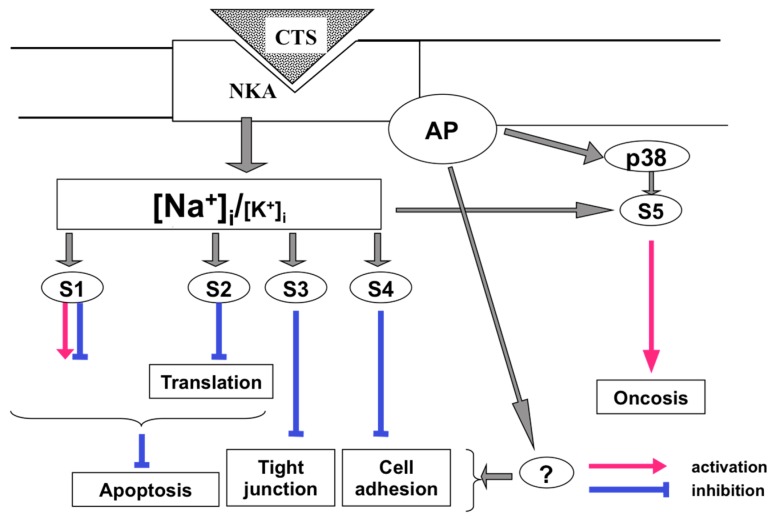
Non-canonical cellular responses triggered by high doses of CTS. Elevation of the [Na^+^]_i_/[K^+^]_i_ ratio triggered by inhibition of the Na^+^,K^+^-ATPase (NKA) by CTS or K^+^-free medium affect transcription and translation via unknown [Na^+^]_i_ and/or [K^+^]_i_ sensors S1 and S2, respectively, that, in turn, leads to inhibition of apoptosis in rodent cells expressing α1R-Na^+^,K^+^-ATPase. In epithelial cells, elevation of the [Na^+^]_i_/[K^+^]_i_ ratio attenuates tight junction and cell adhesion via monovalent ion sensors S3 and S4, respectively. Interaction of CTS with the Na^+^,K^+^-ATPase also leads to conformation transition and interaction with diverse adaptor proteins (AP), including Src, PI3K and InsP_3_R. In cells expressing α1S-Na^+^,K^+^-ATPase, it results in the activation of p38 MAPK and the oncotic mode of cell death. Unlike the suppression of apoptosis, K^+^-free medium does not trigger oncosis, suggesting that the increased [Na^+^]_i_/[K^+^]_i_ ratio contributes to this signaling pathway via the hypothetical sensor S5. The role of Na^+^_i_,K^+^_i_-independent signals in cellular responses triggered via adaptor proteins (AP) and unknown intermediates (?) should be examined further.

The data considered in our mini-review show that several non-canonical cellular responses such as gene transcription and translation, disruption of tight junction, cell adhesion and inhibition of apoptosis in cells expressing α1R-Na^+^,K^+^-ATPase were observed in the presence of high concentrations of CTS, as well as in K^+^-free medium. These observations strongly suggest that these cellular responses are mediated by elevation of the [Na^+^]_i_/[K^+^]_i_ ratio (Figure 9). Importantly, elevation of the [Na^+^]_i_/[K^+^]_i_ ratio might be triggered under diverse physiological and pathophysiological conditions, including sustained excitation of neuronal cells [177], skeletal muscle exercising [178] and hypoxia [179]. What is the molecular origin of [Na^+^]_i_ and [K^+^]_i_ sensors distinct from known membrane-bound transporters involved in the regulation of these cellular responses?

Side-by-side with Na^+^_i_/K^+^_i_-mediated responses, CTS trigger cell type-specific signaling cascades via conformational transitions of the Na^+^,K^+^-ATPase α-subunit and its interaction with Src, PI3K and InsP_3_R (Figure 7 and Figure 8). Recent studies demonstrated the CTS-specific pattern of conformational transitions of the purified Na^+^,K^+^-ATPase [131]. Does this phenomenon contribute to the distinct impact of glycosides and bufadienolides on Src-, PI3K- and InsP_3_R-mediated signaling and downstream cellular responses?

Unlike inhibition of apoptosis in cells expressing α1R-Na^+^,K^+^-ATPase, oncosis detected in several types of cells expressing α1S-Na^+^,K^+^-ATPase is triggered by high concentrations of CTS, but not by Na^+^,K^+^-ATPase inhibition in K^+^-free medium. What is the molecular origin of Na^+^_i_,K^+^_i_-mediated and -independent signal(s) contributing to this mode of cell death (Figure 9)? What is the molecular mechanism of Na^+^,K^+^-ATPase activation and attenuation of the [Na^+^]_i_/[K^+^]_i_ ratio seen in several types of cells treated with low concentrations of CTS? What is the relative impact of signals triggered by the decreased [Na^+^]_i_/[K^+^]_I_ ratio and Src-, PI3K- and IP3R-mediated pathways in augmented proliferation and membrane trafficking demonstrated in cells treated with low concentrations of CTS? What is relative impact of Na^+^_i_,K^+^_i_-mediated and -independent signaling triggered by endogenous CTS in baseline conditions and under their augmented production seen in hypertension and other volume-expanded disorders [8,9,44,180]? These questions will be addressed in forthcoming studies.

## Abbreviations

COS-7	monkey kidney tissue cell line transformated with a mutant strain of SV40 coding for the wild-type T-antigen
CTS	cardiotonic steroids
E1A-adenovirus	adenovirus early region 1A
GC-2	murine spermatogenic cell line
HASMC	human aortic smooth muscle cells
KB-R4943	inhibitor of the Na^+^/Ca^2+^ exchanger
LLC-PK1	kidney proximal tubule cell line
MASMC	mesenteric artery smooth muscle cells
NHE3	sodium–hydrogen exchanger 3
NT2	human neuron-committed teratocarcinoma cell line
RASMC	rat aortic smooth muscle cells
α1R-Na^+^,K^+^-ATPase	CTS-resistant α1-Na^+^,K^+^-ATPase
α1S-Na^+^,K^+^-ATPase	CTS-sensitive α1-Na^+^,K^+^-ATPase
